# The Multifaceted Role of Astrocyte Connexin 43 in Ischemic Stroke Through Forming Hemichannels and Gap Junctions

**DOI:** 10.3389/fneur.2020.00703

**Published:** 2020-07-31

**Authors:** Zhen Liang, Xu Wang, Yulei Hao, Lin Qiu, Yingyue Lou, Yaoting Zhang, Di Ma, Jiachun Feng

**Affiliations:** ^1^Department of Neurology and Neuroscience Center, The First Hospital of Jilin University, Changchun, China; ^2^Department of Plastic and Reconstructive Surgery, The First Hospital of Jilin University, Changchun, China; ^3^Department of Cardiology, The First Hospital of Jilin University, Changchun, China

**Keywords:** ischemic stroke, connexin 43, astrocyte, gap junction, hemichannel, syncytium

## Abstract

Ischemic stroke is a multi-factorial cerebrovascular disease with high worldwide morbidity and mortality. In the past few years, multiple studies have revealed the underlying mechanism of ischemia/reperfusion injury, including calcium overload, amino acid toxicity, oxidative stress, and inflammation. Connexin 43 (Cx43), the predominant connexin protein in astrocytes, has been recently proven to display non-substitutable roles in the pathology of ischemic stroke development and progression through forming gap junctions and hemichannels. Under normal conditions, astrocytic Cx43 could be found in hemichannels or in the coupling with other hemichannels on astrocytes, neurons, or oligodendrocytes to form the neuro–glial syncytium, which is involved in metabolites exchange between communicated cells, thus maintaining the homeostasis of the CNS environment. In ischemic stroke, the phosphorylation of Cx43 might cause the degradation of gap junctions and the opening of hemichannels, contributing to the release of inflammatory mediators. However, the remaining gap junctions could facilitate the exchange of protective and harmful metabolites between healthy and injured cells, protecting the injured cells to some extent or damaging the healthy cells depending on the balance of the exchange of protective and harmful metabolites. In this study, we review the changes in astrocytic Cx43 expression and distribution as well as the influence of these changes on the function of astrocytes and other cells in the CNS, providing new insight into the pathology of ischemic stroke injury; we also discuss the potential of astrocytic Cx43 as a target for the treatment of ischemic stroke.

## Introduction

Ischemic stroke is caused by the stenosis or occlusion of the cerebral blood supply. It is the most common cerebral vascular disease (contributing to ~80% of strokes) with high morbidity and mortality (1, 2). It was recently reported that ischemic stroke, cardiovascular diseases, and malignant tumors constitute the three major causes of human death (3, 4). Although research into the mechanisms of ischemic stroke injury has made advanced progress in the last few years, effective strategies for ischemic stroke treatment to protect residual neurons by restoring brain perfusion as soon as possible via intravenous thrombolysis and mechanical thrombectomy (5, 6) remain limited.

Among the glial cells in the brain parenchyma, astrocytes are the most abundant and critical (7, 8) and may modulate the homeostasis of the central nervous system (CNS) environment and support the survival of neurons (8, 9). The roles of astrocytes in the pathology of ischemic stroke are double-edged, while they can help maintain the homeostasis of the CNS micro-environment to protect neurons by maintaining ion and pH balance, transporting metabolic substrates, and clearing neuronal waste. Conversely, the inflammatory mediators and excitatory amino acids produced and released by astrocytes might promote the death of neurons (10). Connexin 43 (Cx43), one of the most abundant Cxs in the brain tissue, is essential for astrocytes to exert their various physiological functions by forming gap junctions and hemichannels (11, 12) and its role in the development and progression of ischemic stroke have received increasing attention in recent years (13, 14). However, the currently available data show that the change in astrocytic Cx43 after ischemic stroke and the roles it plays are controversial (15–17). Therefore, in this study, we reviewed the syncytium structures that astrocytes form with other cells (astrocytes, neurons, and oligodendrocytes), and the change in astrocytic Cx43 expression and distribution after stroke as well as how these changes influence the functions of astrocytes and the neuro–glial syncytium, subsequently regulating ischemic injury. The delineation of the roles of astrocytic Cx43 in ischemic stroke could help elucidate the initiation and spread of inflammation and neuronal damage after ischemic stroke, which might provide some new targets for the treatment of ischemic stroke.

## The Structure, Distribution, and Physiological Functions of Cx43

Cxs in the CNS are important membrane proteins of a family that consist of 21 members that can form gap junctions and hemichannels. Eleven of these Cxs are expressed in the adult mammalian brain and are distributed differently on glial cells and neurons in the CNS (Table 1) (31–33). Among these Cxs, astrocytic Cx43 is the most widely expressed and studied in the CNS, playing essential roles in the communication between astrocytes and other cells or with the extracellular milieu, as they form gap junction channels or hemichannels (34).

**Table 1:** Cellular distribution of connexins expressed by glia and neurons in the adult mammalian central nervous system.

**Cell type**	**Connexins**	**Gap junctions with astrocytic Cx43**	**References**
Astrocytes	Cx26, Cx30, Cx43	Cx43/Cx43	(18, 19)
Neurons^*^	Cx30.2, Cx31.1, Cx32, Cx36, Cx40, Cx45, Cx50	Cx36/Cx43	(20–23)
Oligodendrocytes	Cx29, Cx32, Cx47	Cx47/Cx43	(24, 25)
Microglia	Cx32, Cx36, Cx43		(26–28)
Capillary endothelial cells	Cx37, Cx40, Cx43		(29, 30)

* *The types of connexins distributed on neurons have not been determined*.

### Structure and Distribution of Cx43

Cx43 in the adult mammalian brain, named after its molecular weight of ~43 kDa, belongs to the α-Cx family and consists of 382 amino acids (31, 35, 36). The same as other Cxs, Cx43 contains four transmembrane regions, the intracellular N-terminal and C-terminal and two extracellular loops. The two extracellular loops and the N-terminal are relatively conserved, while the intracellular loop and C-terminus determine the different biological characteristics in different species (37, 38).

Cx43 is the dominant Cx protein in astrocytes and the main component of astrocytic gap junctions and hemichannels (1). Individual Cx assembles into hexamers around a central pore to form transmembrane channels named connexons, also known as hemichannels (39). They can exist as free, no-junctional channels on the astrocytic membrane and form gap junction channels with other hemichannels on the membrane of adjacent astrocytes or other cells (18). Further studies have shown that hemichannels may be homomeric or heteromeric, depending on the Cx composition. Similarly, gap junctions are homotypic if the paired hemichannels contain the same Cxs, and heterotypic if they contain different Cxs (40).

### Hemichannels

Hemichannels are not closed under resting conditions; their opening probability is very low but not zero (41). However, under certain physiological and pathological stimulation conditions (such as the presence pro-inflammatory cytokines, increase in intracellular calcium concentration, and metabolic inhibition), hemichannels might increase their opening probability (42–47). Consequently, activated astrocytic Cx43 hemichannels are critical ion diffusion (including of Ca^2+^, K^+^, Na^+^) from astrocytes to the extracellular milieu as well as for the release of adenosine triphosphate (ATP) and gliotransmitters [including of glutamate (Glu), adenosine, and glutathione], thus forming chemical coupling between cells and the micro-environment through autocrine and paracrine approaches (48–50). The activated hemichannels might play dual roles in the CNS. Recent reports have shown that the opening of hemichannels during resting conditions is involved in basal synaptic transmission and long-term potentiation (51, 52). Other studies have reported that the opening of hemichannels can facilitate the release of D-serine, further enhancing excitatory synaptic transmission in the hippocampus or olfactory bulb (53). However, in pathological situations, the changes in hemichannels may activate inflammatory signaling, damaging the survival of glial cells and producing excitotoxic molecules (54). After ischemic stroke, inflammation followed by ischemia/reperfusion (I/R) injury could activate the astrocytic hemichannels by increasing extracellular Ca^2+^ and inflammatory cytokine release (15). Then, the opened hemichannels could promote the differentiation of microglia to the M1 phenotype, which could produce pro-inflammatory cytokines including tumor necrosis factor-α (TNF-α) and interleukin-1β (IL-1β), consequently triggering the opening of astrocytic hemichannels (39, 55). The vicious cycle caused the uncontrolled release of ATP, Glu, and Ca^2+^ overload could induce increase in the number of abnormally opened hemichannels, which in turn could lead to tissue excitotoxicity, inflammation amplification, and finally irreversible brain damage (56).

### Gap Junction

Astrocytic gap junctions are axially aligned hexamers of connexins, which can connect adjacent cells (57). Thousands of gap junctions clustered into discoid gap junction plaques could further combine with specific protein binding subunits of paired connexons on the membrane of adjacent cells to form the supramolecular gap junction nexus (58). Gap junctions are the major structures of electrical transmission and metabolic and ionic coupling between adjacent cells (41). In contrast to hemichannels, gap junctions are always open under physiological conditions, allowing intercellular communication (59). They allow small molecules, below 1,200 Daltons, to diffuse, including ATP, inositol trisphosphate, and ions (such as K^+^, Na^+^, and Ca^2+^), cyclic nucleotides, and oligonucleotides and small peptides, facilitating metabolites exchange and information communication among astrocytes, neurons, and oligodendrocytes, maintaining the intracellular and extracellular homeostasis (60–62). More importantly, astrocytic gap junction channels also transmit chemical signals and metabolites (glucose and lactate) between glial cells, facilitating the function of neuronal, glial, and vascular tissues (63).

## The Neuron–Glial Syncytium Structure in the Central Nervous System

**Figure 1:**
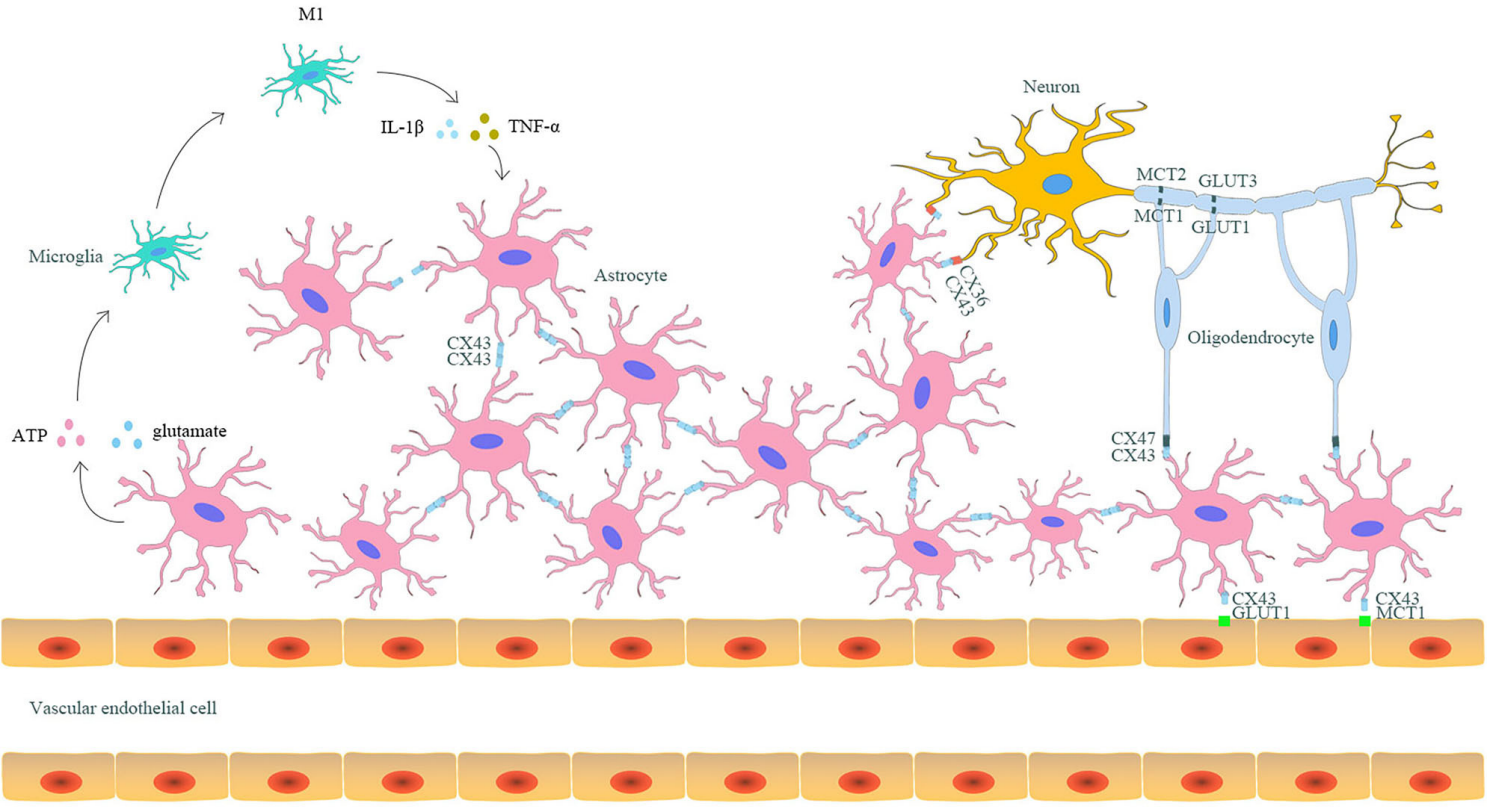
The interaction between astrocytic Cx43 and other parenchymal cells in the CNS. Astrocytes and other parenchyma cells in the CNS form neuro–glial syncytium structures via Cx43/Cx43 gap junctions between astrocytes, Cx43/Cx36 gap junctions between astrocytes and neurons, and Cx43/Cx47 gap junctions between astrocytes and oligodendrocytes. Such structures allow the exchange of metabolites and rapid signal communication, resulting in synergistic response to stimuli. Astrocytic Cx43 is also involved in the capillary-astrocyte-oligodendrocyte-neuron axis and participates in the lactate and glucose transport between capillaries and neurons. Moreover, astrocytes can also indirectly promote the differentiation of microglia to the M1 phenotype through releasing pro-inflammatory mediators via hemichannels. In turn, the differentiated M1 microglia could aggravate the destruction of gap junctions, enhancing injury after ischemic stroke.

As mentioned above, astrocytic Cx43 can both form hemichannels on the cell membrane of astrocytes and form gap junctions with Cx43 or other Cxs on the adjacent astrocytes (18). Considering the hemichannels and gap junctions, astrocytes play a central role in the formation of neuro–glial syncytium structures, where neurons, microglia, oligodendrocytes, and even capillaries could combine to perform various physiological functions (Figure 1) (64).

First, astrocytes can be coupled by Cx43 gap junctions to form syncytium structures, which allows groups of cells to synchronously respond to stimuli (61). The coupled astrocytes can differentiate together during the developmental process (65). They also participate in various physiological processes, including clearing K^+^ from the extracellular space, synthesizing neurotransmitters, propagating calcium waves, balancing Glu and γ-aminobutyric acid, and the immune response (59, 66, 67). However, in ischemic stroke, gap junctions might also act as channels for the transmission of cytotoxic molecules (including of Ca^2+^, excessive ATP, and Glu) from dying astrocytes to their coupled cells, amplifying ischemia-induced brain injury (68).

Apart from astrocyte–astrocyte coupling, astrocytes can also couple with neurons and oligodendrocytes through Cx43 (11, 69). Various studies have confirmed that neurons mainly express Cx36, Cx45, and Cx32, all of which can serve as hemichannels (20, 21). Among these Cxs, Cx36 on neurons can couple with astrocytic Cx43, thus forming Cx43/Cx36 heterotypic gap junctions, which facilitate direct metabolic and electrical communication between astrocytes and neurons (20, 70). Additionally, the existence of Cx43/Cx36 gap junctions can combine neurons into the syncytium network. Under physiological conditions, astrocytes can provide energetic substrates (glucose, lactate, citrate, and glutamine) for neurons. Furthermore, Cx32, Cx47, and Cx29 are primarily expressed on oligodendrocytes (24, 25). Among these Cxs on oligodendrocytes, Cx47 participates in the formation of gap junctions with astrocytic Cx43 (19). Recently, studies have revealed that astrocyte–oligodendrocyte gap junctions are essential for CNS myelination and homeostasis (71). Some studies have found that astrocytes can deliver glucose and lactate to oligodendrocytes through gap junctions, which is essential for the survival of neuronal axons (72, 73). Additionally, heterotypic Cx43/Cx47 gap junctions have been shown to mediate the spatial buffering of K^+^ and the bi-directional transmission of Ca^2+^ between astrocytes and oligodendrocytes (74). The loss of Cx43/Cx47 gap junctions might disrupt the spatial buffering of K^+^, subsequently leading to myelin swelling and axonal degeneration (75, 76).

Although Cxs expressed on capillary endothelial cells (mainly Cx 37, Cx40, and Cx43) can also form hemichannels and gap junctions (29, 30), there is no evidence that astrocytes establish direct contact with endothelial cells, which might account for the obstruction of the basal lamina between these two cell types (77). Astrocytes can attach to capillaries through their end-feet (78, 79). Under normal conditions, capillary endothelial cells can take up blood-borne glucose and lactate by glucose transporter 1 and monocarboxylate transporter (MCT) 1, respectively, which then diffuse through gap junction channels between adjacent endothelial cells. Both glucose and lactate are eventually taken up by the astrocytic end-feet via MCT4 and Cx43 hemichannels, respectively, or released to the extracellular space (80, 81). Thus, glucose and lactate can be transported through astrocytes and their gap junctions with neighboring astrocytes to reach relatively distant areas (82). Furthermore, astrocytes can transfer lactate and glucose to oligodendrocytes through the heterotypic Cx43/Cx47 gap junction channels between them (80). Finally, the lactate in oligodendrocytes can be transported to neuronal axons, inducing axonal degeneration, while the glucose can supply energy for neurons (72, 73).

Taken together, astrocytic Cx43 places astrocytes to a central position in neuron–glial syncytium structures to enroll neurons, oligodendrocytes, microglia, and even capillaries in the CNS into this network, facilitating these cells and structures to respond to changes in the CNS micro-environment, thus maintaining the stability of the CNS milieu and regulating the development, differentiation, and function of neurons.

## Change in Astrocytic Cx43 in Ischemic Stroke

After ischemic stroke, various types of cells in the CNS, including neurons, glial cells, and vascular endothelial cells, sustain different degrees of damage. Astrocytes could be activated and proliferate after ischemic stroke, which is known as reactive astrogliosis (81, 83). Reactive astrogliosis is a type of multistage and pathology-specific reaction, which represents a series of alterations that occur in astrocytes in response to any insult to the CNS (84). At the earlier stage of ischemic stroke, reactive astrocytes can seal the damaged area (85), maintain the balance of the micro-environment, provide nutrients for the neurons, reduce the excitatory toxicity of amino acids, and activate local immune reactions (86). These processes can promote the remodeling of surrounding structural tissues, avoiding secondary damage to neurons (87). However, at the later stage of ischemic stroke, excessive proliferation of reactive astrocytes can change the axon regeneration micro-environment to restrain axonal growth (88), form glial scars, and inhibit the information communication of neurons (89), which can suppress the recovery of nerve function.

Cx43 on astrocytes is an important mediator of CNS ischemic injury, and change in Cx43 expression and distribution has been associated with the outcome of ischemic injury (59). The change in Cx43 expression in the CNS after ischemic stroke remains controversial and depends on ischemia severity, regions, and phase. Cx43 immunoreactivity (Cx43-ir) in the hippocampus and striatum could increase under mild to moderate ischemic conditions (90). However, further studies have indicated that there is an area of reduced Cx43-ir surrounded by a zone of increased Cx43-ir following severe ischemia (66). Another study found that there was a transient downregulation of Cx43 mRNA on day 1 and then upregulation on day 7 after ischemic stroke (91). However, some studies have revealed that there is no significant change in the total amount of Cx43 in *in vitro* and *in vivo* hypoxia models of astrocytes (92). Apart from change in expression, change in Cx43 distribution has also been reported in ischemic stroke. A recent study showed that Cx43 decreased on the astrocytic plasma membrane, whereas it increased in the cytoplasm after ischemic stroke (93). In addition, the reorganization Cx43 gap junctions was also confirmed by immuno-electron microscopy (94).

**Figure 2:**
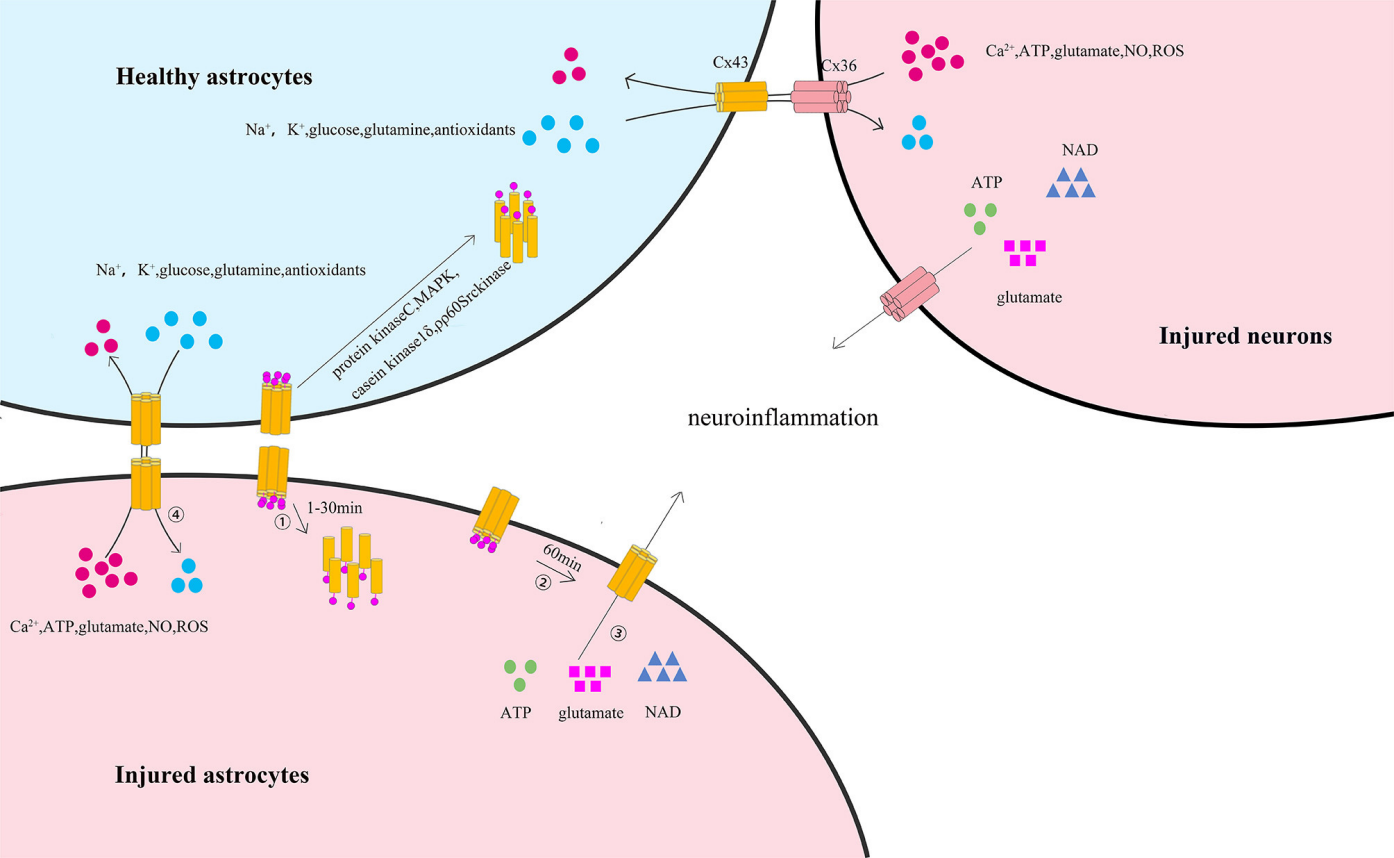
The phosphorylation of astrocytic Cx43 C-terminal and its influence on astrocytes and neurons after ischemic stroke. ① At the early stage of ischemic stroke, astrocytic membrane Cx43, which is involved in the formation of gap junction channels and hemichannels, is phosphorylated at the C-terminal (predominantly from 1 to 30 min) via several protein kinases including protein kinase C, MAPK, pp60Src kinase, and casein kinase 1δ. This process leads to the internalization of membrane Cx43 to the cytoplasm and the degradation of gap junctions. ② At the later stage of ischemic stroke, the remaining astrocytic membrane Cx43 is dephosphorylated (predominantly after 60 min), increasing the permeability of hemichannels. ③ Thus, resulting in the release of ATP, glutamate, and nicotinamide adenine dinucleotide from the cytoplasm of injured astrocytes or injured neurons to the extracellular space, initiating or enhancing neuroinflammation through the remaining gap junctions. ④ In coupled astrocytes, healthy astrocytes could transfer essential ions and metabolites (including Na^+^, K^+^, glutamine, antioxidants, and glucose) to injured astrocytes by Cx43/Cx43 gap junction channels or injured neurons by Cx43/Cx36 gap junction channels and protect the injured astrocytes. In turn, harmful ions and metabolites (including Ca^2+^, ATP, glutamate, NO, and ROS) might also be transferred from injured astrocytes or injured neurons to healthy astrocytes, thus spreading the death wave.

The C-terminal of astrocytic Cx43 has critical roles in regulating astrocytic functions. Various studies have indicated that the phosphorylation status of the astrocytic Cx43 C-terminal is an important mediator modulating the gap junction channels and hemichannels after ischemic stroke, thus influencing the functions of astrocytes and neurons (Figure 2) (95). It has been reported that the C-terminal of astrocytic Cx43 could be phosphorylated after ischemic stroke via several protein kinases including protein kinase C (96), mitogen-activated protein kinase (MAPK) (97), pp60Src kinase (98), and casein kinase 1δ (99), inducing Cx43 internalization, further contributing to the uncoupling process of astrocytes (100, 101). Interestingly, other studies have found that *in vitro* hypoxia may lead to the dephosphorylation of the C-terminal of astrocytic Cx43, accompanied by the uncoupling of astrocytes (101, 102). This controversy arises because the phosphorylation and dephosphorylation of Cx43 as well as astrocytic uncoupling all occur within a short period after ischemia. Further studies have revealed that astrocytic coupling was significantly reduced by 77% after 15 min of hypoxia, 92% after 30 min of hypoxia, and 97% after 1 h of hypoxia, while subsequent substantial Cx43 dephosphorylation was observed at 30 min or 60 min after hypoxia. In addition, a greater quantity of dephosphorylated Cx43 was observed at 60 min than at 30 min. Moreover, dephosphorylated Cx43 became predominant after 60 min. Subsequent studies have also found that phosphorylated Cx43 remained preponderant from 1 min until 30 min after hypoxia, and the level of preserved astrocytic coupling was 34% after 30 min of hypoxia with addition of phosphatase inhibitors to hypoxic astrocytes (101, 103). These studies indicated that the gap junction uncoupling process of astrocytes lies between the phosphorylation and dephosphorylation of Cx43 and might be the result of the phosphorylation of Cx43 and the cause of Cx43 dephosphorylation on hemichannels. However, the precise phosphorylation and dephosphorylation sites of the astrocytic Cx43 C-terminal remain undetermined. Márquez-Rosado et al. revealed that phosphorylation and dephosphorylation occurred at the serine 325/328/330/365/368 (104) site, while Freitas-Andrade et al. demonstrated that phosphorylation and dephosphorylation of serine 255/262/279/282 (105) also occurred. It is unknown whether the phosphorylation and dephosphorylation of astrocytic Cx43 after stroke occurred at the same site and whether the phosphorylation and dephosphorylation of different Cx43 sites might trigger different degrees of Cx43 degradation. Additionally, Cx43 cysteine residues of S-Nitrosylation have been observed in *in vitro* ischemic models induced by nitric oxide (NO) (106). Other *in vitro* studies have found that astrocytic Cx43 was S-Nitrosylated in cultured astrocytes treated with NO for 50 min and that S-Nitrosylated Cx43 could increase the numbers and opening probability of hemichannels (107).

Meanwhile, *in vitro* studies have confirmed that astrocytic Cx43 C-terminal dephosphorylation may increase the opening of hemichannels (108, 109). Previous studies have used astrocytes cultured in *in vitro* ischemia to study the activity of astrocytic Cx43 gap junctions and hemichannels. Among such studies, it has been found that Cx43 gap junctions between dying astrocytes remained functional under ischemic conditions, although Cx43 gap junction coupling decreased (101, 110), signifying that intercellular communication can still occur via astrocytic Cx43 gap junctions under ischemic conditions. Although the opening hemichannels may disrupt the electrochemical and metabolic gradients across the plasma membrane, studies have proven that the remaining gap junctions can protect dying astrocytes to some extent by transferring ions and essential metabolites from healthy astrocytes to dying ones with open hemichannels (107). In contrast, dying astrocytes can also transfer harmful ions and metabolites to neighboring healthy ones with open hemichannels via the remaining gap junctions, which can again cause the death of their neighbors (111). This wave-like spread of the death process is much similar to the extension of infarct regions under ischemic conditions (108, 112, 113).

## Effects of Astrocytic Cx43 in Ischemic Stroke

### Functions of Astrocyte–Astrocyte Coupling

Coupled astrocytes in the astrocytic network share the same fate in ischemic stroke, while the uncoupled ones do not (61, 114). Gap junction communication could positively regulate astrocytic activation and proliferation (115, 116). In permanent right middle cerebral artery occlusion (MCAO) models of Cx43^+/−^ and Cx43^+/+^ mice, Cx43^+/−^ mice showed a significantly larger infarct size but in a smaller area of astrogliosis than did Cx43^+/+^ mice (63), indicating that astrocytic Cx43 gap junctions indeed display a vital role in astrocyte activation and cytotoxic-molecule removal, thus facilitating neuronal survival. Moreover, the astrocytic network formed by gap junctions could also provide energy substrates (glucose and lactate) to neurons (63). Besides, studies with oxygen glucose deprivation (OGD) and MCAO models have found that astrocytic Cx43 is important for astrocytic integrity and stability by activating either astrocytic Cx43 gap junctions or hemichannels (117, 118). Astrocytic Cx43 gap junctions can protect astrocytes by permitting the exit of toxic molecules [including Ca^2+^, excessive ATP, Glu, NO, and radical oxygen species (ROS)] out of the injured astrocyte and the entrance of neuroprotective metabolites (including Na^+^, K^+^, glutamine, antioxidants, glucose) into the astrocytes under ischemic conditions (119, 120). However, if too many toxic molecules are transferred into healthy astrocytes, beyond their bearing load, astrocytic injury might spread to the adjacent astrocytes. Metabolites released by Cx43 hemichannels can also act on the neighboring astrocytes (121). In ischemic stroke, the internalization of astrocytic Cx43 induced by the phosphorylation of the C-terminal might contribute to the uncoupling of astrocytes, thus reducing the mutual support of astrocytes and aggravating their injury (99). In addition, the opening of hemichannels induced by the dephosphorylation of astrocytic Cx43 could promote the release of inflammatory mediators, increasing neuroinflammation after ischemic stroke (108, 109).

### Functions of Astrocyte–Neuronal Coupling

Recent studies have found that astrocytes could protect neurons by producing glutathione to exert anti-oxidant effects, reducing inflammatory media, enlarging the gap junctions, regulating energy metabolism, inhibiting apoptosis, up-taking excitatory amino acids, inducing cerebral ischemic tolerance in response to ischemic preconditioning, and other processes (122, 123). Accumulating evidence indicates that the amount of Cx43/Cx36 gap junctions decreases due to Cx43 internalization after ischemic stroke, which reduces the transportation of neurotransmitters and metabolites between astrocytes and neurons (100). However, aerobic metabolism and ATP production declined while toxic ions and molecules, such as Ca^2+^, glutamate, ROS, and NO, accumulated in damaged neurons after ischemic stroke (124). These toxic metabolites may be released by hemichannels and transmitted from damaged neurons to healthy astrocytes via Cx43/Cx36 gap junctions, thus reducing the load of neurons and activating astrocytes (125). The activated astrocytes could release pro-inflammatory cytokines and chemokines (80), which could in turn lead to changes in the neuronal functions that affect behavior, mood, and cognitive abilities (126). Furthermore, pro-inflammatory cytokines decreased the gap junctions between astrocytes and increased the opening of neuronal Cx36 and astrocytic Cx43 hemichannels, finally causing an increase in the release of ATP, Glu, prostaglandins, and NO (127–129). These molecules are toxic to adjacent cells, which may amplify the inflammation and cause secondary damage to distant cells, leading to tissue excitotoxicity and irreversible brain damage (56, 130).

### Functions of Astrocyte–Oligodendrocyte Coupling

Studies have reported that the existence of astrocytic Cx43 is necessary for the functions of oligodendrocytes (71, 131). The loss of astrocytic Cx43, which forms hemichannels and gap junction channels on the astrocytic membrane, could disrupt the Cx43/Cx47 gap junctions, which are harmful for the transmission of ions and nutrients between astrocytes and oligodendrocytes (132). Studies have found that Cx43 displays multiple metabolic and signaling roles in astrocytes, which can affect oligodendrocytes independently of gap junctions (133). Studies with astrocytic Cx43-deficient mice have revealed that Cx47 was not stabilized and its amount was strongly decreased because of internalization and degradation (131), which lead to the diffusion of Cx47 away from the oligodendrocytic cell membrane, aggravating the post-ischemic inflammatory response and myelin loss of oligodendrocytes (134, 135).

### Interaction of Astrocytes and Microglia

Although microglia express Cx36, Cx32, and Cx43 (26, 136), gap junctions between microglia and astrocytes have not been observed. In addition, extracellular ATP released from astrocytes accounting for the opening of hemichannels after ischemic stroke could activate the purine ionotropic receptors on microglia (including P2X_4_R and P2Y_12_R), thereby promoting the differentiation of microglia to the M1 subtype (56, 137). Activated microglia could secret TNF-α and IL-1β, which could further aggravate the inflammation (138). Activated microglia could inhibit gap junction communication and downregulate Cx43 expression in astrocytes through the release of TNF-α and IL-1β by mix culturing of astrocytes and activated microglia induced by lipopolysaccharides. Interestingly, Meme et al. found in subsequent experiments that activated microglia treated with lipopolysaccharides were four times more efficient than untreated microglia in inhibiting gap junction communication and Cx43 expression (139). We assume that the reason for this diversity might be that TNF-α and IL-1β are mainly secreted by activated, rather by untreated, microglia. More importantly, TNF-α and IL-1β released by activated microglia could increase astrocytic-hemichannel activity. The increased hemichannels can continuously release ATP, further activating the microglia (38, 128). Thus, abnormally opened astrocytic hemichannels, secondary ATP release, and activated microglia-mediated neuroinflammation may complement each other, leading to a vicious cycle of continuously aggravating post-ischemic tissue damage.

However, the interactions of astrocytes and microglia exert neuroprotective effects under some pathological conditions such as traumatic brain injury (TBI). Microglia in the injury core could be activated after TBI and then release ATP and inflammatory cytokines, and ensuing downregulation of the P2Y1 receptor could then transform the astrocytes to a neuroprotective phenotype (140). Then, reactive astrogliosis would occur in the peri-injured region and accelerate neuroprotective astrocytic scar formation, thus relieving inflammation (141).

### Interaction of Astrocytes and Capillaries

The interaction between astrocytes and capillaries is quite meaningful for the energy supply of neurons by astrocytic MCT4 and capillary GLUT. In addition, the lactate in capillaries could also be transported to astrocytes by astrocytic Cx43 hemichannels and capillary MCT1, and further delivered to neuronal axons, inducing axonal degeneration (80, 82). Recent studies have revealed that the opening of astrocytic Cx43 hemichannels could increase after ischemic stroke, contributing to substantial lactate diffusion to astrocytes and neuronal axons (93, 142). Studies have also found that the overexpression of astrocytic MCT4 under hypoxia provides more rapid transmission of glucose, facilitating the energy supply of neurons (82). Furthermore, astrocytes were considered to produce vasoactive factors in response to neuronal activity by their hemichannels, causing rapid and localized changes in cerebral blood flow after ischemic stroke (143, 144). Thus, astrocytes may nourish neurons by controlling the glucose and lactate availability through the regulation of blood flow (81).

## Therapies and Applications

Nowadays, an increasing number of studies focus on the significance of Cx43 for irreversible injury after ischemic stroke. The influence of the expression and distribution of Cx43 and the block of hemichannels might affect the outcomes of ischemic stroke. Several astrocytic Cx43 targeted reagents or drugs have been considered to be potential therapeutic in cerebral I/R injury (145).

Leptin is a multifunctional hormone secreted by adipocytes and could regulate food intake and energy metabolism (146). Deng et al. found that leptin could also suppress the elevation of Cx43 expression via the ERK/MAPK signaling pathway in MCAO mice *in vivo*, further alleviating cerebral I/R injury. In the same study, the authors also demonstrated that leptin could inhibit Cx43 elevation in SY5Y and U87 cells, thus reducing Glu release by inhibiting the function of Cx43 hemichannels and decreasing cell death in *in vitro* OGD models (147). Carbenoxolone (CBX) is another widely used hemichannel blocker in diverse pathological processes in the brain. It has been proven that CBX can inhibit the release of ATP, further inhibiting or reversing the activation of microglia (148). The latest studies have revealed that CBX can switch the differentiation of activated microglia from M1 to M2, thus providing effective neuroprotection after ischemic stroke (93, 149). Apart from directly blocking hemichannels, a recent study revealed that CBX may influence Cx43 hemichannels and gap junctions by indirect mechanisms such as phosphorylation or internalization of Cx43 subunits (150).

Cx43 mimetic peptides, including Gap 19, Gap 26, Gap 27, peptide 5, and L2 peptide, can also serve as Cx43 hemichannel blockers, further reducing I/R injury (151, 152). In these Cx43 mimetic peptides, Gap26 and Gap27 were found to not only inhibit the opening of hemichannels after ischemic stroke in neonatal rats, but also to modulate gap junction communication due to their poor specificity to Cx43 hemichannels at high concentrations and/or following prolonged exposure (153, 154). Indeed, Gap26 has also been confirmed to protect the heart of rats against myocardial ischemic injury induced by ligation of the left anterior descendent (LAD) by selective inhibition of hemichannels at low concentrations of Gap26 (0.5 μM) (155). Gap27 could also reduce the myocardial infarct size in rat LAD models (156). Interestingly, the function of peptide 5 was shown to be concentration dependent; it could block hemichannels at 5 μM, while blocking gap junctions at 500 μM (157). The L2 sequence is located on the cytoplasmic loop of Cx43, and the Gap19 sequence is a nine amino acid stretch within the L2 domain (158). The L2 peptide can stabilize the open state of gap junctions while blocking hemichannels (158). Slightly different from the L2 peptide, Gap19 blocks hemichannels while not influencing gap junctions on short exposure (30 min) but slightly inhibits them on longer exposures (24–48 h) (158). In addition, studies with Gap19-treated mice after ischemic stroke induced by MCAO found that Gap19 could attenuate the white matter infarct volume by suppressing the expression of Cx43 and of inflammatory cytokines (TNF-α and IL-1β) as well as inhibiting Toll-like receptor 4 pathway activation; experiments with *in vitro* OGD ischemic stroke models have also confirmed these results (55). Furthermore, treating mouse MCAO models with Gap19 also reduced the myocardial infarct size by blocking Cx43 hemichannels (159). More importantly, Gap19 appeared to be more effective than Gap26/27 in reducing the myocardial infarct volume after heart ischemia (160), possibly because Gap26/27 is less selective to Cx43 gap junctions and hemichannels and it can inhibit channels composed of Cxs other than Cx43 (161).

## Conclusion

Astrocytes are the central cells in the neuron–glial syncytium, which can combine parenchyma cells in the CNS into a whole to rapidly and synchronously respond to stimuli by forming Cx43/Cx43 gap junctions with other astrocytes, Cx43/Cx36 gap junctions with neurons, Cx43/Cx47 gap junctions with oligodendrocytes, and indirect interactions with microglia. This markedly large network ensures better between-cell communication and increases tolerance to ischemia. The gap junctions between astrocytes and other cells have an important role in substance and metabolite exchange and cell communication. In ischemic stroke, the phosphorylation of astrocytic Cx43 might lead to the uncoupling of gap junctions between astrocytes and other parenchymal cells, reducing the direct communication between these cells. The subsequent dephosphorylation of Cx43 on hemichannels activates the opening of hemichannels, promoting the release of various pro-inflammatory mediators and toxic molecules, such as ATP and Glu. Meanwhile, astrocytic Cx43 could also become S-Nitrosylated after ischemic stroke, increasing the numbers and opening probability of hemichannels. However, the change in astrocytic Cx43 expression and distribution remains controversial and might depend on ischemia duration, region, and severity. Besides, how ischemia induces change in astrocytic Cx43 expression and distribution and the underlying mechanism also need to be delineated.

The destruction of the neuro-glial syncytium structure resulting from the uncoupling of corresponding gap junctions might weaken the mutual support between astrocytes and other cells, and the increase in hemichannel numbers caused by the uncoupling of gap junctions and permeability caused by the dephosphorylation of Cx43 might enhance and spread neuroinflammation and aggravate injury after ischemic stroke. Based on these theories, astrocytic Cx43 might be a potential target for ischemic stroke treatment. However, Cx43 participates both in the formation of gap junctions and of hemichannels. The former is more likely to play a beneficial role, whereas the latter is more likely to be deleterious in ischemic stroke; accordingly, agents that simply target Cx43 might not have the expected therapeutic effect. Therefore, it might be more meaningful to explore agents that can specifically block hemichannels or promote the maintenance of gap junctions. What is more important is that the currently available research that has focused on Cx43-associated agents was conducted using animal and cell models; whether these agents act protectively in patients with ischemia and their safety in the clinic require further exploration.

## Author Contributions

ZL contributed to the design of the study and wrote the first draft of the manuscript. YH contributed to the design of the study. LQ, YL, and YZ drew the pictures. XW, DM, and JF contributed to the editing of the manuscript. All authors contributed to the article and approved the submitted version.

## Conflict of Interest

The authors declare that the research was conducted in the absence of any commercial or financial relationships that could be construed as a potential conflict of interest.

## Abbreviations

ATP: Adenosine triphosphate
CBX: carbenoxolone
CNS: central nervous system
Cx: connexin
Glu: glutamate
GLUT: glucose transporters
IL-1β: interleukin-1β
LAD: left anterior descendent
MAPK: mitogen-activated protein kinase
MCAO: middle cerebral artery occlusion
MCT: monocarboxylate transporter
NO: nitric oxide
OGD: oxygen glucose deprivation
ROS: radical oxygen species
TNF-α: tumor necrosis factor-α.
